# Activation likelihood estimation identifies brain regions activated during puncturing at *Hegu* in healthy volunteers: A meta-analysis

**DOI:** 10.3389/fnins.2022.1084362

**Published:** 2022-12-22

**Authors:** Zhen Gao, Mengjie Cui, Jing Zhang, Laixi Ji

**Affiliations:** ^1^Experimental Management Center, Shanxi University of Traditional Chinese Medicine, Jinzhong, Shanxi, China; ^2^Affiliated Hospital of Shanxi University of Traditional Chinese Medicine, Taiyuan, Shanxi, China

**Keywords:** acupuncture, *Hegu*, LI 4, ALE, neuroimaging

## Abstract

**Background:**

*Hegu* is the most commonly used acupoints for pain relief. Recently, several functional neuroimaging studies have been performed on acupuncture at *Hegu* in healthy volunteers, but these studies have yielded diverse findings. Therefore, there is an urgent need to understand the brain response characteristics of acupuncture at *Hegu*.

**Methods:**

Neuroimaging studies on acupuncture at *Hegu* published before October 2022 were collected from PubMed, Web of Science, Google Scholar, Embase, and CNKI (China National Knowledge Infrastructure) databases, and were screened by strict inclusion and exclusion criteria. The extraction of brain coordinates was performed by two independent researchers, and the results were analyzed using activation likelihood estimation (ALE) analysis based on quantitative coordinates.

**Results:**

In total, 338 studies were searched, of which 19 studies were included in the final analysis after a rigorous double-blind screening review. Activation likelihood estimation showed that postcentral gyrus in the left brain were activated, whereas the anterior cingulate in the left brain and superior temporal gyrus in the right brain were deactivated.

**Conclusion:**

Acupuncture at *Hegu* in healthy volunteers did not reveal specific brain regions. This finding implies that organismal status of the study subjects may have an important impact on the effect of acupoints.

**Systematic review registration:**

[https://www.crd.york.ac.uk], identifier [CRD42020197296].

## Introduction

*Hegu* is one of the most commonly used acupoints, located at the dorsal part of the hand and the midpoint of the radial side of the second metacarpal bone (Chae et al., 2007; Zhang et al., 2013). Numerous clinical studies have demonstrated the efficacy of *Hegu* in treatment of shoulder pain, carpal tunnel syndrome, toothache and other painful diseases. However, the underlying biological mechanism of action remains unclear, and further studies are necessary (Sheng and Chang, 1960; Wang et al., 2006; Napadow et al., 2007; Lu, 2011; Asadi et al., 2015).

In the absence of clinical conditions, healthy individuals are ideal subjects for determining brain activity during acupuncture stimulation (Huang et al., 2022). To date, neuroimaging techniques have been used to identify brain patterns that activate or deactivate during acupuncture at *Hegu* in healthy individuals. Research has found evidence that a large number of brain regions are involved, but due to a variety of discrepancies in research settings (e.g., study design, etc.), no consensus has been reached. For example, a previous study found that the analgesic effect of acupuncture at *Hegu* is related to the pregenual anterior cingulate and hippocampal (Claunch et al., 2012). However, *Hegu* has also been shown to evoke activation in the thalamus, basal ganglia and cerebellum, as well as in the left putamen (Gu et al., 2015). In addition, identifying brain regions that change activity when acupuncture *Hegu* helps to determine the effect of acupuncture on the central nervous in humans. Therefore, there is an urgent need to collate previous evidence and uncover the altered brain activity associated with acupuncture at *Hegu*.

Activation likelihood estimation (ALE) has developed as an important meta-analysis approach for synthesizing neuroimaging data (Tillisch et al., 2011). It has been widely used in the study of acute sleep deprivation, insomnia, and other disorders (Tahmasian et al., 2018a; Javaheripour et al., 2019). ALE can maximize consistency of locating information, among quantitative studies, and can also minimize heterogeneity of the analytical method. With the aid of ALE, this study aimed to perform a meta-analysis of the results of previous studies to obtain reliable conclusions about the functional changes in brain regions induced by acupuncture at *Hegu*, and to help elucidate the mechanisms of *Hegu*.

## Methods

### Search strategy and study selection

We searched PubMed, Web of Science, Google Scholar, Embase, and CNKI (China National Knowledge Infrastructure) database for relevant studies published up to October 2022. Our search contains the following search terms: (*Hegu* OR LI 4) AND (voxel-based morphometry OR morphometric OR VBM OR neuroimaging OR functional neuroimaging OR functional magnetic resonance imaging OR functional MRI OR fMRI OR positron emission tomography OR PET) AND (acupuncture therapy OR acupuncture OR electroacupuncture OR electro-acupuncture OR acupoint* OR meridian*). Studies that met the following criteria were included: (1) analyzed only on healthy volunteers with acupuncture at *Hegu*; (2) Acupuncture includes body acupuncture, manual acupuncture, warm acupuncture, ear acupuncture, plum blossom needling, fire needling and electrical acupuncture; (3) reported results were on whole-brain scans rather than area-of-interest scans; (4) reported results were in normalized spatial coordinates, including Montreal neurological institute (MNI) or Talairach coordinates; and (5) the study adopted a task design. Conversely, those that met the following criteria were excluded: (1) transcutaneous electrical nerve stimulation, acupressure and laser stimulation as acupuncture interventions; (2) conference papers, reviews and animal experiment studies; or (3) only reported individual subject rather than group data. Data extraction for the included studies was independently performed by two reviewers, based on a data collection list that included effective sample size, age and gender distribution, handiness, acupuncture method, acupuncture laterality, task paradigms, and reported peak coordinates (x, y, z) in the standard atlas (Talairach or MNI). A third reviewer double-checked the data when necessary to ensure consistency.

### Activation likelihood estimation

Activation likelihood estimation is a method that uses voxel coordinates to locate and analyze functional brain regions. This approach has been previously utilized to integrate reported coordinates from different studies (Turkeltaub et al., 2012; Tahmasian et al., 2018b). Through the method, consistency of spatial positions among various studies can be maximized while minimizing subjectivity of the analysis method. All coordinates published in Talairach space were transformed into MNI space, using the Ginger ALE software (BrainMap Ginger ALE 3.0.2; Research Imaging Center, University of Texas Health Science Center at San Antonio), while individual coordinates were modeled by a 3-D Gaussian probability distribution. Thereafter, we created a modeled activation map (MA map) after combining the probability distributions of all foci, then incorporated the MA maps to produce the final ALE map, which reflected the likelihood convergence of results across all studies (Eickhoff et al., 2012). The ALE map was assessed against null-distribution of random spatial association, using histogram integration, at a statistical significance of *p* < 0.05 family wise error in cluster level (cFWE) to correct for multiple comparisons (Eickhoff et al., 2017; Müller et al., 2018). Finally, we adopted the anatomical image overlay program Mango (Creators, Jack L. Lancaster and Michael J. Martinez)^1^ to illustrate the meta-analysis results using MNI coordinates.

### Quality assessment

The quality of the included studies was assessed using an 11-point checklist that has been used in previous meta-analyses, which focused on population demographic characteristics, scanner parameters, and methodological details (Shepherd et al., 2012; Du et al., 2014; Cheng et al., 2020). Two reviewers independently conducted the quality assessment of the study, and any disagreements were resolved by a third reviewer. The meta-analysis has been registered in the PROSPERO International Prospective Register of Systematic Reviews of the University of York (No. CRD42020197296).

## Results

**FIGURE 1:**
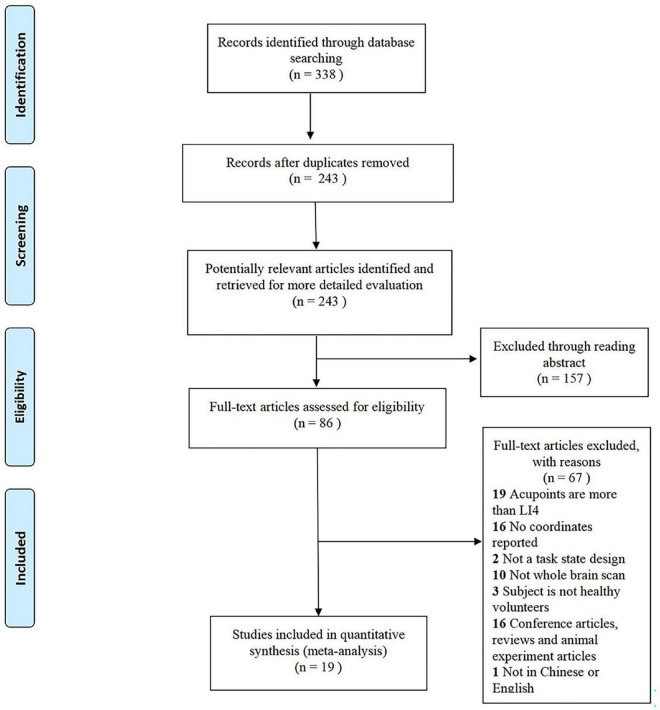
The flow diagram for studies included in the present meta-analysis.

In this meta-analysis, from 338 retrieved studies, 19 studies and 399 subjects (216 females) were eligible to be included in this meta-analysis (Figure 1). These 19 studies were all functional magnetic resonance imaging (fMRI), including 16 block designs and 3 non-repeat event-related design (Table 1). All included studies had a mean quality score of 9.34, out of a possible total of 11, indicating that they were of high quality.

**TABLE 1:** Studies entered into the meta-analysis are listed based on the year of publication.

References	Sample size	Handedness	Acupoint	Imaging modality	MRI scanner	Processing software	Stimulation	*deqi*	Experimental design
Chen, 2018	76	Right	Unilateral	fMRI	3.0-T Siemens	LC Model	MA	Yes	Block design
Wang, 2017	10	Right	Bilateral	fMRI	3.0-T GE	AFNI	MA	Yes	Block design
Wang et al., 2015	80	Right	Bilateral	fMRI	1.5-T Siemens	AFNI	MA	Yes	Block design
Gu et al., 2015	12	Right	Unilateral	fMRI	3.0-T Siemens	SPM8	EA	Yes	Block design
Chae et al., 2015	17	Right	Unilateral	fMRI	3.0-T Siemens	SPM8	MA	Yes	Block design
Yang et al., 2014	20	Right	Unilateral	fMRI	1.5-T Siemens	AFNI	MA	NA	Block design
Liu, 2014	8	Right	Unilateral	fMRI	3.0-T Philips	NA	EA	NA	Non-repeat event-related design
Claunch et al., 2012	46	NA	Unilateral	fMRI	1.5-T Siemens	AFNI	MA	Yes	Block design
Chen et al., 2011	13	Right	Unilateral	fMRI	1.5-T GE	AFNI	MA	Yes	Block design
Asghar et al., 2010	17	Right	Unilateral	fMRI	3.0-T GE	FAL	MA	NA	Block design
Tang et al., 2009	12	Right	Unilateral	fMRI	1.5-T GE	SPM2	MA	Yes	Block design
Tong, 2008	8	Right	NA	fMRI	1.5-T Siemens	SPM2	MA	NA	Non-repeat event-related design
MacPherson et al., 2008	17	Right	Unilateral	fMRI	3.0-T GE	FSL	MA	NA	Block design
Zhang et al., 2007	11	Right	Unilateral	fMRI	1.5-T Philips	SPM2	MA	Yes	Block design
Wang et al., 2007	9	NA	EA	fMRI	1.5-T Siemens	SPM2	EA	Yes	Block design
Li et al., 2006	18	Right	Unilateral	fMRI	1.5-T Siemens	SPM2	MA	Yes	Non-repeat event-related design
Li et al., 2005	6	Right	Unilateral	fMRI	1.5-T Siemens	SPM2	MA	Yes	Block design
Hou et al., 2002	8	Right	Unilateral	fMRI	1.9-T GE	NA	MA	Yes	Block design
Kong et al., 2002	11	Right	Unilateral	fMRI	1.5-T GE	AFNI	EA	Yes	Block design

fMRI, functional magnetic resonance imaging; NA, data not available; MA, manual acupuncture; EA, electrical acupuncture.

**FIGURE 2:**
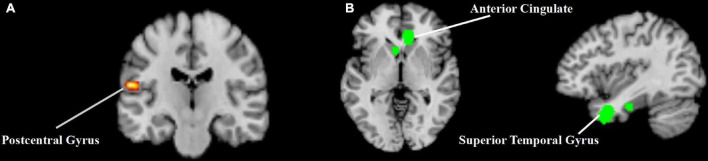
The results of the activation likelihood estimation (ALE) analysis from acupuncture at *Hegu*. **(A)** The activation likelihood estimation (ALE) analysis result of the activation patterns of acupuncture at *Hegu*. **(B)** The activation likelihood estimation (ALE) analysis result of the deactivation patterns of acupuncture at *Hegu*.

The meta-analysis of the activation patterns of acupuncture at *Hegu* included 19 studies, 407 subjects and 307 foci (Figure 2A and Table 2). Analysis showed that acupuncture stimulation caused an activation pattern in the left postcentral gyrus.

**TABLE 2:** Activation likelihood estimation (ALE) meta-analysis results of acupuncture at *Hegu.*

Analysis	Cluster number	Cluster size (mm^3^)	MNI coordinates			ALE value (10^–3^)	Location			
					
			*x*	*y*	*z*		Hemisphere	Lobe/Sub-lobe	Gyrus/Nucleus	Broadman area
Activation patterns of acupuncture at *Hegu*	1	10,784	–60	–18	18	23.21	L	Parietal lobe	Postcentral gyrus	40
Deactivation patterns of acupuncture at *Hegu*	1	13,848	8	34	0	14.95	R	Limbic lobe	Anterior cingulate	24
	2	10,320	–40	8	–32	13.21	L	Temporal lobe	Superior temporal gyrus	38

L, left; R, right.

The meta-analysis of the deactivation patterns of acupuncture at *Hegu* included 11 studies, 301 subjects and 168 foci (Figure 2B and Table 2). Analysis showed that acupuncture stimulation caused occurrence of common deactivation patterns across various brain regions, such as right anterior cingulate and left superior temporal gyrus.

Since at least 17 studies were required to achieve 80% power for moderate effects (Eickhoff et al., 2016), separate subgroup analyses were not performed for the different experimental designs.

## Discussion

To the best of our knowledge, this study used a voxel-based meta-analysis to provide the first report of brain response characteristics during acupuncture at *Hegu*. The results showed that acupuncture at *Hegu* caused functional activation or deactivation across several non-specific brain regions, including the sensorimotor network and the limbic system.

In this study, postcentral gyrus showed significant signal increases. The results revealed that acupuncture at *Hegu* activated the sensorimotor network, which receives sensory information from the periphery and is critical in bodily sensation and in generating appropriate motor responses, and is the main brain network responsible for pain perception (Mayer et al., 2015). Since acupuncture is an invasive form of mechanical stimulation, when the needle penetrates the body, the acupuncture signal is received by the pain receptors, integrated through the thalamus, and transmitted to postcentral gyrus. The postcentral gyrus serves as the primary somatosensory cortex responsible for receiving nociceptive and proprioceptive sensations from the contralateral body, processing sensory information from the somatosensory areas and participating in the discrimination of the level, location and duration of painful stimuli (Zhang et al., 2014). Although not entirely consistent, the present results and the findings of Chae et al. (2013) confirm that mechanical stimulation caused by the therapeutic tool during needling activates sensorimotor network, which is caused by acupuncture itself. Consistent with previous studies (Hui et al., 2000), this study also found evidence that acupuncture at *Hegu* triggers extensive deactivation of the cerebral cortex, including the anterior cingulate cortex (ACC) and superior temporal gyrus that overlap with the limbic system (Catani et al., 2013; Fulford, 2015). Furthermore, the limbic system has been shown to play an important role in the acupuncture effects of multiple acupoints (Fang et al., 2009). For example, *Zusanli* (ST 36) also deactivates brain regions such as the middle superior frontal gyrus (Xiang et al., 2019) ang ACC (Claunch et al., 2012). Apparently, no specific brain regions were observed in the present study during acupuncture at *Hegu*.

Several previous reviews have explored the modulatory effects of acupuncture on the brain. A systematic review based on acupuncture at *Zusanli* in healthy volunteers showed that brain regions such as superior temporal gyrus, insula, and postcentral gyrus were activated after acupuncture, which is similar to our findings, suggesting similar brain responses at different acupoints (Huang et al., 2022). However, Dhond et al. (2007) analyzed the functional changes of the brain under various pain states, and proposed that certain limbic brain networks may play a specific therapeutic role in the process of acupuncture analgesia. Consistent with Huang et al.’s (2012) study, the presence of this difference suggests that patients and healthy volunteers have different brain responses to acupuncture stimulation. According to traditional Chinese medicine theory, acupoints are considered a dynamic functional area, which can reflect the internal condition of the body (Tan et al., 2019). Under physiological conditions, acupoints are silent and their functions are not manifested or obvious, but under pathological conditions, acupoints are activated and their functional effects can be manifested (Zhu, 2019). Several studies have shown that acupuncture at the same acupoints has significant differences in brain function changes under different organismal states. For example, a study found that compared with healthy subjects, acupuncture in patients with low back pain have increased functional connectivity in brain regions such as the thalamus, brainstem, and insula (Ye et al., 2011). Cho et al. (2013) also proposed that brain signal activations during the same acupuncture were different between the healthy and the stroke patients. All of these brain imaging studies confirmed that functional status is an essential impact factor for cerebral responses to acupuncture stimulation, and specific brain regions are difficult to obtain through healthy subjects. In addition, the application of acupuncture in clinical practice is specific to the pathological rather than the physiological state. Therefore, disease is a better way to study the effects of acupoints.

Task state design is a unique and diverse design approach that analyses activated brain regions by presenting subjects with specific stimuli during scanning. To improve the homogeneity of this meta-analysis, we only included studies with a task state design to improve and this helped in identifying brain patterns associated with acupuncture. In addition, we chose a more rigorous FWE algorithm for brain imaging meta-analysis to make the results more reliable (Eickhoff et al., 2016). However, this study has limitations that need to be addressed. Since the sample sizes of such studies are usually small, we proposed stricter inclusion and exclusion criteria, resulting in a limited sample size for the final meta-analysis. In addition, due to the large number of identical brain regions, we reported the results only for the peak with the largest ALE value.

## Conclusion

Acupuncture at *Hegu* in healthy volunteers resulted in activation or deactivation of the sensorimotor network and the limbic system, did not reveal specific brain regions. The action of acupoints is dynamic and the functional status appears to be an essential impact factor for cerebral responses to acupuncture stimulation. Future studies should focus on the brain’s response to acupuncture treatment under different diseases.

## Author contributions

ZG contributed to the study conception and design, conceived the data analysis strategy, collated and analyzed the data, and drafted the manuscript. MC and JZ acquired the data. ZG, MC, JZ, and LJ discussed, read, and revised the manuscript. All authors approved the publication of this manuscript.

## Footnotes

1
http://rii.uthscsa.edu/mango
