# Supplementary material for: Activation likelihood estimation identifies brain regions activated during puncturing at Hegu in healthy volunteers: A meta-analysis
Source: Front Neurosci. 2022 Dec 22;16:1084362. doi: 10.3389/fnins.2022.1084362 (PMC9813741; doi:10.3389/fnins.2022.1084362)
Supplement: Supplementary file 1 [file Table_1.DOCX]

Table 1 Activation likelihood estimation (ALE) meta-analysis results of MA and EA at *Hegu*

| Analysis | Cluster number | Cluster Size (mm^3^) | MNI coordinates | | | ALE value (10^-3^) | Location | | | |
| --- | --- | --- | --- | --- | --- | --- | --- | --- | --- | --- |
|  |  |  | x | y | z |  | Hemisphere | Lobe/sub-lobe | Gyrus/nucleus | Broadman Area |
| Activation patterns of MA at *Hegu* | 1 | 10736 | -60 | -18 | 18 | 23.21 | L | Parietal Lobe | Postcentral Gyrus | 40 |
|  | 1 |  | -64 | -24 | 24 | 13.42 | L | Parietal Lobe | Postcentral Gyrus | 40 |
|  | 1 |  | -54 | -26 | 24 | 12.59 | L | Parietal Lobe | Inferior Parietal Lobule | 40 |
|  | 1 |  | -58 | -20 | 28 | 12.32 | L | Parietal Lobe | Inferior Parietal Lobule | 40 |
|  | 1 |  | -34 | -16 | 14 | 11.37 | L | Sub-lobar | Claustrum |  |
|  | 1 |  | -46 | -20 | 22 | 11.31 | L | Sub-lobar | Insula | 13 |
|  | 1 |  | -64 | -10 | 28 | 9.16 | L | Parietal Lobe | Postcentral Gyrus | 3 |
|  | 1 |  | -60 | -20 | 0 | 6.16 | L | Temporal Lobe | Superior Temporal Gyrus |  |
| Deactivation patterns of MA at *Hegu* | 1 | 14968 | 8 | 34 | 0 | 14.94 | R | Limbic Lobe | Anterior Cingulate | 24 |
|  | 1 |  | 6 | 20 | -16 | 13.61 | R | Limbic Lobe | Anterior Cingulate | 25 |
|  | 1 |  | -6 | 28 | -10 | 13.51 | L | Limbic Lobe | Anterior Cingulate | 24 |
|  | 1 |  | -2 | 24 | -18 | 11.87 | L | Limbic Lobe | Anterior Cingulate | 32 |
|  | 1 |  | -8 | 34 | -14 | 10.77 | L | Limbic Lobe | Anterior Cingulate | 32 |
|  | 1 |  | -2 | 16 | 12 | 10.26 | L | Sub-lobar | Caudate |  |
|  | 1 |  | 12 | 34 | -26 | 9.82 | R | Frontal Lobe | Medial Frontal Gyrus | 25 |
|  | 1 |  | 0 | 14 | -14 | 9.61 | L | Limbic Lobe | Anterior Cingulate | 25 |
|  | 1 |  | -6 | 14 | 10 | 9.03 | L | Sub-lobar | Caudate |  |
|  | 1 |  | 8 | 36 | -18 | 8.54 | R | Limbic Lobe | Anterior Cingulate | 32 |
|  | 1 |  | -10 | 22 | -12 | 8.42 | L | Sub-lobar | Caudate |  |
|  | 1 |  | -6 | 16 | -2 | 7.80 | L | Sub-lobar | Caudate |  |
|  | 1 |  | 4 | 40 | -20 | 7.75 | R | Frontal Lobe | Medial Frontal Gyrus | 11 |
| Deactivation patterns of EA at *Hegu* | 1 | 29320 | 50 | 30 | -2 | 6.85 | R | Frontal Lobe | Inferior Frontal Gyrus | 45 |
|  | 1 |  | 38 | 12 | -6 | 6.55 | R | Sub-lobar | Claustrum |  |
|  | 1 |  | 40 | 10 | -32 | 6.28 | R | Temporal Lobe | Superior Temporal Gyrus | 38 |
|  | 1 |  | 26 | 6 | -22 | 6.20 | R | Limbic Lobe | Parahippocampal | 34 |
|  | 1 |  | 28 | 12 | -30 | 6.04 | R | Temporal Lobe | Superior Temporal Gyrus | 38 |
|  | 1 |  | 48 | 24 | -28 | 3.95 | R | Temporal Lobe | Superior Temporal Gyrus | 38 |
|  | 2 | 27232 | -32 | 18 | -4 | 6.53 | L | Sub-lobar | Claustrum |  |
|  | 2 |  | -34 | -14 | -26 | 6.27 | L | Limbic Lobe | Parahippocampal |  |
|  | 2 |  | -38 | 10 | -30 | 6.21 | L | Temporal Lobe | Superior Temporal Gyrus | 38 |
|  | 2 |  | -24 | 4 | -20 | 6.19 | L | Limbic Lobe | Parahippocampal | 34 |
| MA, manual acupuncture; EA, electrical acupuncture; L, left; R, right. | | | | | | | | | | |

Table 2 PRISMA-A checklist

| **Subjects** | **PRISMA for Acupuncture** | | **Reported on page #** |
| --- | --- | --- | --- |
| ***Title*** |  |  |  |
| **Title** | 1^*^ Identify the report as a systematic review, meta-analysis, or both; if applicable, state the specific type of acupuncture treatment, such as manual acupuncture or electroacupuncture. | | # 1 |
| ***Abstract*** |  |  |  |
| **Structured summary** | 2^†^ Provide a structured summary including, as applicable: background; objectives; data sources; study eligibility criteria, participants, and interventions; study appraisal and synthesis methods; results limitations; conclusions and implications of key findings; systematic review registration number. | | # 2 |
| ***Introduction*** |  |  |  |
| **Rationale** | 3^*^ Describe the rationale for what is already known about acupuncture for the target condition in the background; if applicable, state what is already known about the specific types of acupuncture to be studied, and describe whether there is any difference of the effects among different types of acupuncture. | | # 3 |
| ***Methods*** |  |  |  |
| **Protocol and registration** | 5^†^ Indicate if a review protocol exists, if and where it can be accessed (e.g., web address), and, if available, provide registration information including registration number. | | # 6 |
| **Eligibility criteria** | 6^†^ Specify study characteristics (e.g., PICOS, length of follow-up) and report characteristics (e.g., years considered, language, publication status) used as criteria for eligibility, giving rationale.  6a.1^‡^ Describe the diagnostic criteria of the target condition in Western medicine.  6b^‡^ Describe the types of acupuncture to be included, such as traditional acupuncture, electroacupuncture, or fire acupuncture. | | # 4-5 |
| **Information sources** | 7^*^ Describe all sources of information (e.g., databases with dates of coverage, contact with study authors to identify additional studies) in the search, and report the date of the last search. If applicable, report the databases or complementary search methods for acupuncture or traditional medicine. | | # 4 |
| **Search** | 8^*^ Present full electronic search strategy for at least one commonly used database (e.g. MEDLINE), including any limits used, such that it could be repeated. If applicable, include the full search strategy for at least a Western and a traditional medicine database for each systematic review where both were used. | | # 4 |
| **Study selection** | 9^†^ State the process for selecting studies (i.e., screening, eligibility, included in systematic review, and, if applicable, included in the meta-analysis). | | # 5 Figure 1 |
| **Data collection**  **process** | 10^†^ Describe method of data extraction from reports (e.g., piloted forms, independently, in duplicate) and any processes for obtaining and confirming data from investigators. | | # 5 |
| **Data items** | 11^*^ List and define all variables for which data were sought (e.g., PICOS, funding sources) and any assumptions and simplifications made; describe data items about details of acupuncture interventions and controls (**e.g.,** sham acupuncture) referring to TIDieR when applicable. | | # 4-5 |
| ***Results*** |  |  |  |
| **Study selection** | 17^†^ Give numbers of studies screened, assessed for eligibility, and included in the review, with reasons for exclusions at each stage, ideally with a flow diagram. | | Figure 1 |
| **Study characteristics** | 18* For each study, present characteristics that were extracted (e.g., study size, PICOS, follow-up period) and provide the citations of the included studies. Summarize details of the acupuncture intervention for each study in a table referring to TIDieR**.**  18a^‡^ Describe details of “De-qi” after acupuncture reported in the included studies. | | # 7-8 |
| ***Discussion*** |  |  |  |
| **Limitations** | 25^†^ Discuss limitations at study and outcome level (e.g., risk of bias), and at review level (e.g., incomplete retrieval of identified research, reporting bias). | | # 13 |
| **Conclusions** | 26^†^ Provide a general interpretation of the results in the context of other evidence, and implications for future research. | | # 13 |
| ***Funding*** |  |  |  |
| **Funding** | 27^†^ Describe sources of funding for the systematic review and other support (e.g., supply of data); role of funders for the systematic review. | | # 14 |
